# Cross Modal Perception of Body Size in Domestic Dogs (*Canis familiaris*)

**DOI:** 10.1371/journal.pone.0017069

**Published:** 2011-02-16

**Authors:** Anna M. Taylor, David Reby, Karen McComb

**Affiliations:** Mammal Vocal Communication and Cognition Research, School of Psychology, University of Sussex, Brighton, United Kingdom; Indiana University, United States of America

## Abstract

While the perception of size-related acoustic variation in animal vocalisations is well documented, little attention has been given to how this information might be integrated with corresponding visual information. Using a cross-modal design, we tested the ability of domestic dogs to match growls resynthesised to be typical of either a large or a small dog to size-matched models. Subjects looked at the size-matched model significantly more often and for a significantly longer duration than at the incorrect model, showing that they have the ability to relate information about body size from the acoustic domain to the appropriate visual category. Our study suggests that the perceptual and cognitive mechanisms at the basis of size assessment in mammals have a multisensory nature, and calls for further investigations of the multimodal processing of size information across animal species.

## Introduction

Body size is a critical attribute that has been linked to resource holding potential, fighting ability, long-term survival and reproductive prospects for males and females in many species [1], [2] and therefore its assessment is central to social and sexual interactions. There is increasing evidence that size assessment in the acoustic domain might be as important as size assessment in the visual domain [3]–[5]. In particular, vocal tract resonances (formants) are a reliable acoustic correlate of caller body size [3]–[5]. In several species, including domestic dogs, formants are directly predictive of vocal tract length, which in turn is predictive of overall individual body size [6]. Several experiments suggest that animals attend to formants in conspecific signals [7]–[9]. However, less attention has been given to how size information in the acoustic domain might be integrated with size information in the visual domain.

Habituation-discrimination experiments have shown that receivers perceive size-related differences in formant dispersion [10]–[14], but the results of such paradigms only demonstrate perceptual ability. Some studies investigating the functional value of formant perception have found that the behavioural responses of receivers vary consistently according to the apparent body size of playback stimuli simulating potential rivals or mates [8], [9], [15], suggesting that at least in the auditory channel, size-related acoustic variation has both perceptual and functional relevance. However, a systematic multi-channel investigation is required to show that beyond just perceiving and reacting to size-related acoustic variation, animals can perform a functionally relevant integration of size information in the acoustic domain with size information in the visual domain. Ghazanfar and colleagues [16] have previously demonstrated under laboratory conditions that untrained rhesus monkeys can attribute resynthesised coo calls to images of adult (larger) or subadult (smaller) conspecifics. While it remains unclear whether the monkeys attributed coos on the basis of size-related variation in formant frequencies, or on the basis of the age-related variation as these are confounded in this experimental design [16], the results of this study did suggest that non-human primates have the ability to cross-modally integrate information on age-related size variation. This raises two important questions. Firstly, is this spontaneous cross-modal integration a primate-only adaptation, or rather a common ability across mammals? Secondly, can mammals integrate caller size information within, rather than across age categories?

Here we use a preferential looking paradigm to assess whether the domestic dog, a non-primate mammal that has previously been shown to attend to formants in conspecific growls in a manner consistent with the ability to assess size [9], is capable of spontaneously matching size information in the growls of adult conspecifics with corresponding visual size categories. Animals are tested in their natural, ecologically valid environment and are not provided with any training or reward. Preferential looking paradigms have been successfully used in human infants [17], [18] and nonhuman primates [16], [19]–[20] to test the ability of subjects to match an auditory stimulus to a corresponding visual stimulus. Where cross-modal ability exists, it is interpreted as an indication that subjects perceive information across the acoustic and visual channels as being categorically associated [17]–[23] and there is growing interest in the neural bases of this multisensory integration in animals [24] and humans [25]. Due to the potential methodological limitations of using video displays with non-human subjects [26], we here use different sized stuffed dog models for the visual stimuli (a Jack Russell terrier and a German Shepherd).

We therefore test whether dogs are able to integrate the acoustic size information contained in resynthesised growls in which the formant dispersion has been scaled to be typical of either small or large dogs with size-matched visual stimuli. Based on previous experiments using preferential looking, we predict that when presenting a resynthesised growl to a domestic dog that is within visual range of two differently sized dog models, the subject will look at the size-matched model more often and for a longer duration than at the unmatched alternative. We also investigate the direction of the first look.

## Results

**Figure 1:**
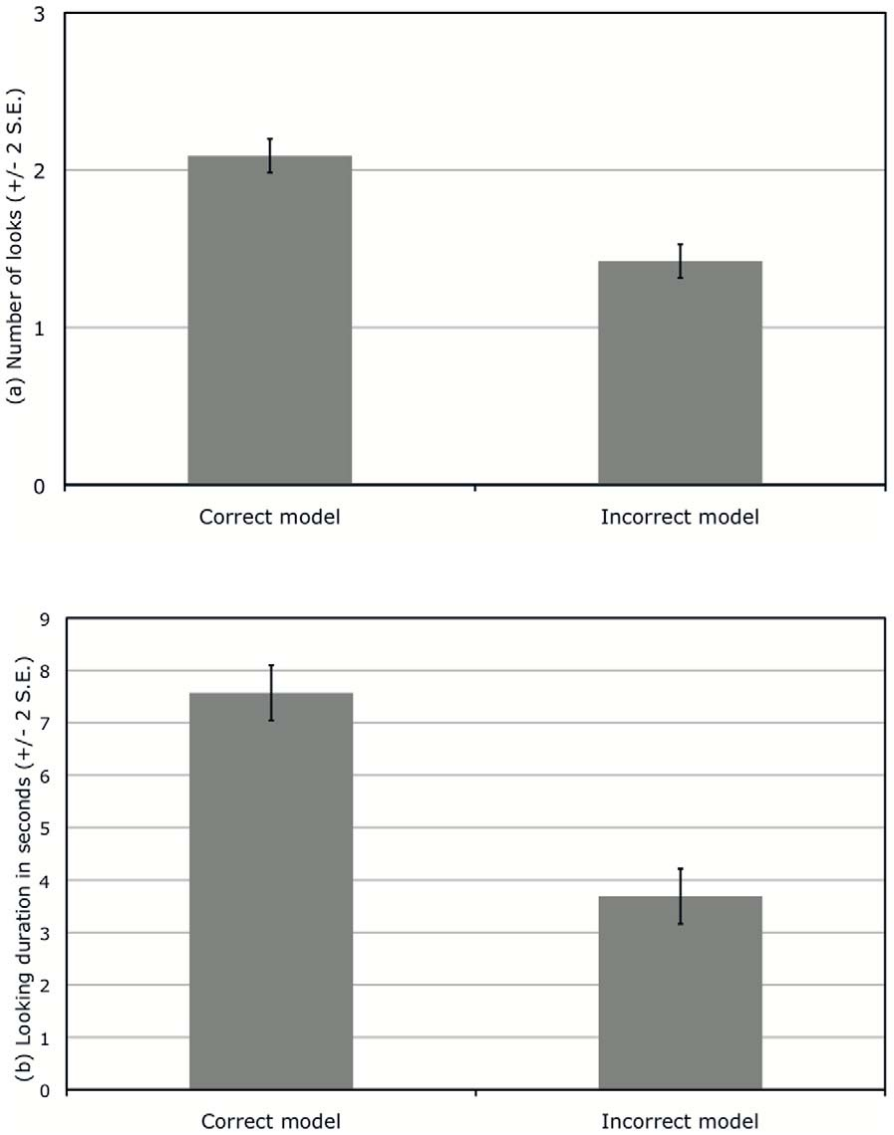
Number of looks (a) and looking duration (b) at the correct and incorrect models.

Overall, subjects looked at the correct model a mean 2.1 times and at the incorrect model a mean 1.4 times, as illustrated by Figure 1(a). The linear mixed model analysis showed that this difference was statistically significant (F_(1,75)_  = 8.464, p = 0.001), and that number of looks was not affected by subject sex (F_(1,75)_ = 0.012, p = 0.913, n.s.), playback condition (F_(1,75)_  = 0.897, p = 0.345, n.s.) or the side of the correct model (F_(1,75)_  = 0.012, p = 0.913, n.s.). The co-variates, age and dog weight did not account for any of the variance in the number of looks, but there was a marginal effect of order of presentation on number of looks (F_(1,75)_  = 3.993, p = 0.049); specifically, fewer looks were given on second presentation than on first presentation. This was most likely due to habituation to the experimental set-up. There were no significant interaction effects between any of the variables.

Moreover, subjects looked at the correct model for a mean 7.6 seconds and at the incorrect model for a mean 3.7 seconds, as illustrated by Figure 1(b). Again, this difference was found to be significant (F_(1,75)_  = 13.029, p = 0.001), demonstrating that subjects looked at the correct model for a significantly longer duration than at the incorrect model. Looking duration was not affected by subject sex (F_(1,75)_  = 0.497, p = 0.483, n.s.), playback condition (F_(1,75)_  = 0.616, p = 0.434, n.s.), side of the correct model (F_(1,75)_  = 0.665, p = 0.418, n.s.) or order of presentation (F_(1,75)_ = 0.026, p = 0.875, n.s.). Finally, the co-variates, age and dog weight did not account for any of the variance in the looking duration, and there were no significant interaction effects between any of the variables.

While on the first stimulus presentation, only 58% of subjects looked at the correct model first (Binomial test, p = 0.430, n.s.), on the second stimulus presentation, 68% of subjects looked at the correct model first (Binomial test, p = 0.038). This result suggests that on the second presentation, subjects were significantly more likely to look at the correct model first, presumably because by then the subjects were more familiar with the experimental set-up and the visual reference of the models. The duration of the first look, however, was not affected by presentation order (F_(1,75)_  = 0.331, p = 0.567, n.s.), playback condition (F_(1,75)_  = 0.217, p = 0.643, n.s.), side of the correct model (F_(1,75)_ = 1.742, p = 0.191, n.s.) or whether the look was correct or not (F_(1,75)_ = 2.452, p = 0.122, n.s.).

## Discussion

Our results provide unequivocal evidence that domestic dogs have the ability to match size information in the acoustic domain with corresponding size information in the visual domain in an ecologically valid environment. Dogs exposed to growls in which the formant dispersion was resynthesised to be typical of either a small dog or a large dog looked at a size-matched model significantly more often and for a significantly longer duration than at a size-unmatched model. This ability was independent of model (small or large dog), playback condition (small or large acoustic stimulus), side of the correct model (left or right), or playback exemplar. It was also unaffected by subject age or sex. The order of presentation was found to have a marginally significant effect on the number of correct looks and on whether the first look was correct or not. However in both cases this is likely to reflect habituation to the experimental procedure and does not detract from the main results of the experiment.

It should be noted that as the model dogs were from two different breeds, our results are specific to size differences in the visual domain as represented by the combination of these two particular breeds. However, since the audio stimuli came from a wide range of animals of different breeds and sizes, and since each growl exemplar was re-synthesised to both the small and large variant, size was the only parameter that was consistently common between the presented audio stimuli and one of the two visual models. The observed results cannot, therefore, be attributed to uncontrolled confounding factors associated to these specific breeds. Indeed, it seems highly unlikely that the ability of dogs to assess size differences in the visual domain is limited to the German Shepherd/Jack Russell terrier breed contrast, and we are thus confident that these results can be generalised to other breeds presenting comparable size differences.

Further work is now required to investigate the range of size variation that dogs are able discriminate, both in the auditory domain and in the visual domain. Due to the wide range of morphological and size variation across dog breeds, the extent or limitations of this ability will play a substantial role in the coordination of intra-specific interactions in the current socio-ecological environment of domestic dogs. The attribution of body size from a vocal signal could for example be important in social contexts similar to that set up for our experiment, where dogs encounter two unfamiliar conspecifics of different sizes but hear only one growl. As growls are primarily an aggressive signal [15], the rapid cross-modal attribution of a growl to a size-matched caller could be vital during group interactions.

As previously noted, while it seems likely that some species may use visual cues to assess body size [1], [2], the evolution of deceptive visual signals (e.g. piloerection in aggressive displays; [27]) as well as highly ritualised behaviours such as parallel walking in red deer stags [28] or rearing up in elephant seals [29] indicates that visual size assessment in dynamic interactions is not necessarily reliable or straightforward. Indeed these ritualised behaviours are likely to have evolved to enable or facilitate size assessment by standardising visual displays. Moreover, visual perception is directional and can be affected by distance and visibility (dense vegetation, darkness), providing a relative adaptive advantage to acoustic perception in assessment situations. Although acoustic signals can be subject to attenuation, amplitude fluctuations or reverberation during propagation, at medium ranges they remain robust to degradation [5], [30] and are thus an excellent channel of communication over relatively long distances and in conditions of reduced visibility [30], [31] or when making a functional decision about whether to escalate an agonistic encounter.

The integration of acoustic information on body size is unlikely to have evolved as a consequence of domestication or the ensuing morphological variation across domestic dog breeds. Rather, we hypothesise that it is likely to have been selected for across a range of mammalian (or more generally of vertebrate) species and further studies on different species are now warranted to investigate this claim. More generally, cross-modal integration of sensory cues is unlikely to be specific to body size, but rather to be functional for many different types of information. Indeed, our findings are consistent with the recent growing body of literature reporting the ability of several mammalian species to categorically integrate different types of information across the acoustic and visual channels such as emotional state [21], age-related size [16] or individual identity [23], [31]. Multisensory integration should be highly functional in a social context as it provides animals with a coherent perceptual experience [30]–[34]. Experimental paradigms that tap into abilities for cross-modal integration of sensory cues can thus be applied to study how animals categorise different types stimuli in their environment.

In sum, while it is clear from previous experiments that several mammalian species, including domestic dogs, have the ability to perceive size-related acoustic variation in growls and furthermore that they respond to playback stimuli in ways that are functionally consistent with size assessment, we have here demonstrated an additional level of perceptual and cognitive ability, namely the integration of size information across the auditory and visual domains.

## Materials and Methods

### Subjects

Forty adult dogs of different breeds were used for the study. The subjects were recruited when their owners responded to an online advertisement for the experiment. The selection criteria for subject animals was that they had to be healthy adults (older than one year) with no known sight or hearing problems and no known aggression to humans. None of the dogs had participated in any previous vocal communication or behaviour research. All subjects were tested in August and September 2008 in one of three indoor testing locations in the East Sussex area (University of Sussex in Falmer, Raystede Rescue Centre in Ringmer, PAWS Dog Training in Willingdon).

### Recordings and resynthesis of the playback stimuli

The growls used as the basis for the playback stimuli were recorded from ten dogs of different breeds, ages and sex between October 2005 and August 2006. The dogs were recorded using a Marantz PMD670 digital audio recorder with a Sennheiser MKH 416 directional microphone. All growls were recorded in the same social context, in which the experimenter entered the dog's home and stared the animal in the eyes to elicit defensive growling [15], [35].

To create the playback stimuli, the recorded growls were manipulated with Praat version 4.4.32 (Boersma & Weenink, The Netherlands) using a PSOLA (Pitch-Synchronous Overlap Add)-based algorithm. This algorithm is able to shift the formant frequencies of acoustic signals while leaving all other acoustic parameters unchanged. The resulting stimuli are thus identical in all ways (including duration, amplitude contour, fundamental frequency) other than their formant dispersion [7], [15], [35]. Each growl was resynthesised twice: once to have a formant dispersion of 1500 Hz simulating a vocal tract length of 11.7 cm that corresponds to a very small dog of approximately 6 kg, and a second time to have a formant dispersion of 850 Hz simulating a vocal tract length of 20.6 cm that corresponds to a large dog of approximately 40 kg [33]. We thereby created “small dog” and a “large dog” variants of each growl that were identical in all acoustic parameters other than formant dispersion (Audio S1) - these resynthesis parameters were selected to match the size of the visual models as determined by average breed measurements [35]. Each trial consisted of two identical growls (from the same size variant) repeated with a 2 second interval. Previous research has shown domestic dogs respond to such resynthesised growls in the same way as to natural growls [8]. Finally, the playback stimuli were peak amplitude normalised to 75% to standardise their intensity.

### Experimental set-up and playback procedure

**Figure 2:**
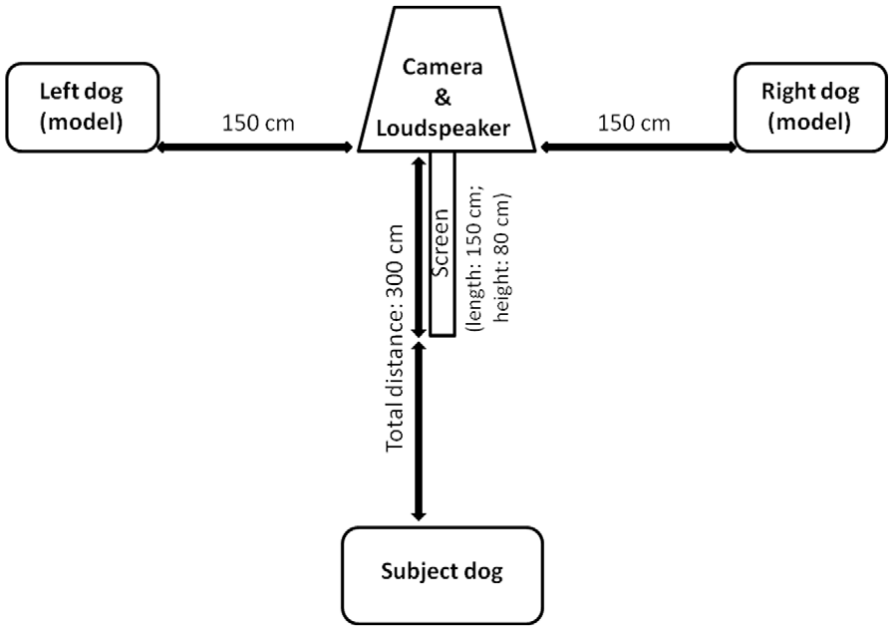
Experimental set-up, showing the layout of the testing area and distances between subject, models and loudspeaker.

The experiment was conducted in August and September 2008 using a cross-modal ‘preferential looking’ design [16]–[18], [21]–[22]. The design was developed on the basis of pilot trials conducted in February and March 2008 on twenty subjects (who did not take part in the final trials) and the final experimental setup is schematically illustrated in Figure 2. An Anchor Liberty 6000HIC loudspeaker was located at 300 cm in front of the designated subject area and two dogs model were placed facing the subjects at 150 cm from either side of the loudspeaker (there was thus a total of 340 cm between the two models, with the loudspeaker in the middle). The models were of two different breeds: a German shepherd dog (standing at approximately 60 cm) hired from a professional taxidermist and a Jack Russell terrier (standing at approximately 30 cm) purchased for the purpose of this experiment. These breeds are both utility dogs that are comparable in type in that they do not show any morphological abnormalities caused by selective breeding. Finally, a 150×80 cm woodchip screen was used to create a visual barrier between the models as pilot trials found that this facilitated the coding of looks given by the subjects by accentuating the directionality of gazing.

**Figure 3:**
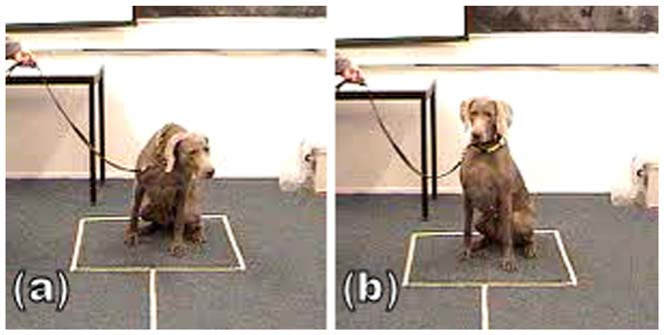
Subject sitting in the designated testing area, looking at the left model (a) and the right model (b).

The subjects were placed either sitting or standing in a clearly designed area (Figure 3). Neither the experimenter nor the owner were in their field of vision during the experiment in order to minimise unconscious cueing. They remained on the loose lead (not pulled tense) throughout the experiment and were always handled by their owner. The owners were naïve to the purpose of the experiment and were instructed to remain behind the dog and to look down at their own hands for the duration of the experiment so as not to inadvertently give any cues to their dogs. After being positioned for the experiment, subjects were given 20 seconds to visually familiarise themselves with the models and settle down. They were not permitted to approach the models for olfactory exploration. After the familiarisation period, the playback experiment started.

The design was counterbalanced so that each model was on the left or right an equal number of times and so that each subject was exposed to a large and a small audio variant. Small and large variants presentations were played in a pseudo-randomised fashion, so that each pairing of small and large variants occurred only once and the subjects never heard the same growl exemplars as a small and a large variant). The stimuli were played at 55 dB (+/- 5 dB), which was the mean amplitude of growling registered in our recording sample as measured by a CEL-414 Precision Impulse Sound Level Meter. Each playback trial lasted 25 seconds (detailed information about coding is in the Video Data section). Finally, the interval between playback conditions was fixed at 60 seconds, as pilot tests indicated that this was the best time to maintain both subject interest and lack of novelty effect. No training or rewards were provided to the dogs.

### Ethical statement

The experiment was designed to replicate an everyday encounter between domestic dogs, a situation that was considered to be familiar to the subjects as they were all socialised with other dogs. The dogs were privately owned and handled by their owner at all times. Because of its observational and non-invasive nature, the experiment did not require a licence under the United Kingdom Home Office regulations concerning animal research and welfare. The study complied with the internal University of Sussex regulations on the use of animals and was approved by the School of Psychology ethics committee. The dogs that took part in this study did not show any behavioural signs of distress at any point during the experiment.

### Video data

The subjects were filmed using SONY Carl Zeiss Vario-Tessar DCR-HC51 handycam with a 16-bit digital PCM stereo sound card and built-in stereo microphone. White masking tape was used to create floor markings that were clearly visible on the video in order for there to be a physical determinant of left and right when coding the videos. Videos were converted to .mov files and coded on a frame-by-frame (0.04 s) basis using the digital video analysis software Gamebreaker version 5.4.48 [36]. A coding protocol was developed with number of correct looks versus incorrect looks and total duration of looking toward the correct model in the 25 seconds following each stimulus presentation.

A “look” was defined as a directional static stare at the model of a minimum of one second. Coding was unambiguous as the models were 300 cm apart in the horizontal plane and floor markers and screen provided a clear visual barrier between the left and right models. The dogs thus had to make fairly large head movements of approximately 45 degrees to look at one or the other model. In addition, the dogs frequently oriented their body posture in the direction of looking. To prevent coding biases, the videos were coded blind in a random order, with the coder unaware which side was correct and incorrect. In addition, 10% of the videos were double-coded by a naïve research assistant and a strong inter-observer reliability correlation validated the coding for both number of looks (Pearson's R^2^_adj_  = 0.92, p<0.001) and duration of looking (Pearson's R^2^_adj_  = 0.96, p<0.001).

### Statistical analyses

Linear mixed models in SPSS 16.0 for Mac OS X (10.4.11) were used to analyse the data. The linear mixed model is particularly suited to experimental designs in which there are repeated and non-repeated elements [15], [35] and is fitting for the current design in which subjects were exposed to two conditions but heard each type of stimulus only once. To control for variance caused by individual differences, subjects were controlled for as a repeated measure with potential random effects. Playback condition, side of the correct model, order of presentation, playback exemplar and subject sex were included as fixed factors. Age and subject weight were included as co-variates. The model also investigated any potential interaction effects between any of the variables. Model fit was verified by the returned AIC score and by the examination of residuals to confirm their homogeneity.

## Supporting Information

Audio S1 **Example of the resynthesised stimuli.** This audio file contains three exemplars of growls, each resynthesised to the small and large variants.
(WAV)
